# Susceptibility of Human Prion Protein to Conversion by Chronic Wasting Disease Prions

**DOI:** 10.3201/eid2408.161888

**Published:** 2018-08

**Authors:** Marcelo A. Barria, Adriana Libori, Gordon Mitchell, Mark W. Head

**Affiliations:** National CJD Research and Surveillance Unit, University of Edinburgh, Edinburgh, Scotland, UK (M.A. Barria, A. Libori, M.W. Head);; National and OIE Reference Laboratory for Scrapie and CWD, Canadian Food Inspection Agency, Ottawa, Ontario, Canada (G. Mitchell)

**Keywords:** prion, chronic wasting disease, protein misfolding cyclic amplification, zoonoses, deer, elk, sheep, reindeer, North America, Europe

## Abstract

Chronic wasting disease (CWD) is a contagious and fatal neurodegenerative disease and a serious animal health issue for deer and elk in North America. The identification of the first cases of CWD among free-ranging reindeer and moose in Europe brings back into focus the unresolved issue of whether CWD can be zoonotic like bovine spongiform encephalopathy. We used a cell-free seeded protein misfolding assay to determine whether CWD prions from elk, white-tailed deer, and reindeer in North America can convert the human prion protein to the disease-associated form. We found that prions can convert, but the efficiency of conversion is affected by polymorphic variation in the cervid and human prion protein genes. In view of the similarity of reindeer, elk, and white-tailed deer in North America to reindeer, red deer, and roe deer, respectively, in Europe, a more comprehensive and thorough assessment of the zoonotic potential of CWD might be warranted.

Chronic wasting disease (CWD) is a fatal contagious prion disease of cervids that is found in the United States, Canada, South Korea, and most recently in Europe (*1*,*2*). The species affected differ in these geographic areas; mule deer (*Odocoileus hemionus*), white-tailed deer (*O. virginianus*), North American elk (*Cervus canadensis*), and moose (*Alces alces*) are most commonly affected in the United States and Canada, and red deer (*C. elaphus*) and sika deer (*C. nippon*) in South Korea also have been affected (*1*,*2*). The first identification of CWD in Europe occurred in 2016 in wild moose (*A. alces*, also known as Eurasian elk) and in a free-ranging reindeer (*Rangifer tarandus,* closely related to the free-ranging caribou of North America), a species not previously known to be affected by CWD in wild or farmed animals (*2*,*3*). CWD is a pressing animal health issue, but whether it might become a human public health issue should also be considered.

Scrapie and bovine spongiform encephalopathy (BSE) are well-characterized animal prion diseases affecting animals of consumption; scrapie affects sheep and BSE cattle. Human prion diseases occur as sporadic, genetic, and acquired forms; the variant form of Creutzfeldt-Jakob disease is zoonotic BSE, acquired through the oral route and having a long incubation period (*4*). In contrast, sheep scrapie is generally considered to be of no or only very low risk to human health, although this possibility has been questioned recently (*5*,*6*). The molecular basis of prion replication is a change in conformation of the normal cellular prion protein (PrP^C^) into the abnormal and misfolded conformer (PrP^Sc^) that is partially protease-resistant (PrP^res^). However, the molecular criteria for predicting zoonotic potential for prions are unclear. Consequently, approaches to understanding the zoonotic risk for prion transmission to humans have been empirical, involving in vivo and in vitro models (*7*). Experimental transmission of mule deer CWD to nonhuman primates showed that squirrel monkeys are susceptible, whereas cynomolgus macaques appear to be resistant (*8*,*9*). Similarly, attempted transmission of deer or elk CWD to transgenic mouse lines expressing human PrP^C^ have largely failed, indicative of a species barrier (*10*–*13*), although this might be overcome in some instances (*14*).

In contrast to these in vivo approaches, we have used protein misfolding cyclic amplification (PMCA) to investigate the molecular compatibility of bovine, ovine, and cervid prions with full-length, glycosylated and glycosylphosphatidylinositol-anchored human prion protein (PrP) (*15*,*16*). We found that scrapie samples failed to convert human PrP^C^ to PrP^res^, whereas cattle BSE converted the human protein efficiently (*15*–*17*). These observations suggest that PMCA can reproduce aspects of cross-species transmission potential and inform assessment of zoonotic risk. The single CWD-affected specimen (from a North American elk) previously available to us was found to be capable of converting human PrP^C^ to PrP^res^ (*15*). Here, we expand on the previous report; analyzing more elk specimens of 2 different genotypes (132 MM, homozygous for methionine at *Prnp* position 132, and 132 ML, methionine–leucine heterozygous at the same position), analyzing white-tailed deer CWD specimens for the first time, and analyzing reindeer that have been experimentally infected with white-tailed deer CWD. The elk ML polymorphism at position 132 of *Prnp* is of particular interest because it corresponds to the methionine–valine (MV) *PRNP* codon 129 polymorphism in humans, which is itself a major genetic susceptibility factor associated with human prion disease (*18*). Although these examples of CWD all derive from North America, the inclusion of white-tailed deer (a relative of European roe deer, *Capreolus capreolus*), North American elk (a near relative of red deer), and, most important, reindeer might help in formulating risk assessments in Europe.

## Materials and Methods

### Animal Tissue

We obtained from the National and OIE Reference Laboratory for Scrapie and CWD (Ottawa, ON, Canada) elk, white-tailed deer, and reindeer frozen brain tissue from CWD-affected animals that had been confirmed positive by using a statutory diagnostic testing regime (Table). Small pieces of tissue were obtained, and we analyzed all for the presence of PrP^res^ by using Western blot, as previously described (*15*). We used transgenic mouse brains produced by gene replacement and expressing physiologic levels of the human PrP of the 3 *PRNP* codon 129 polymorphic genotypes as the PMCA substrate (*20*).

**Table:** Description of cervid CWD specimens used to evaluate the susceptibility of human prion protein to conversion by in vitro conversion analysis*

Species	Common name		Specimen ID	Disease in captive animals or experimental transmission	*Prnp* genotype, known relevant polymorphisms
	North America	Europe			
*Cervus canadensis*	North American or Rocky Mountain elk or wapiti	Closely related to red deer (*Cervus elaphus*)	Elk 0†	Captive	132 MM
			Elk 1	Captive	132 MM
			Elk 2	Captive	132 MM
			Elk 3	Captive	132 MM
			Elk 4	Captive	132 MM
			Elk 5	Captive	132 ML
			Elk 6	Captive	132 ML
*Odocoileus virginianus*	White-tailed deer	Related to roe deer (*Capreolus capreolus*)	WTD 1	Experimental	96 GG
			WTD 2	Experimental	96 GG
*Rangifer tarandus* subspecies	Caribou (free-ranging) or reindeer (captive)	Reindeer (free-ranging and captive)	Reindeer 1	Experimental (transmission of WTD [CWD 96GG])‡	§
			Reindeer 2	Experimental [transmission of WTD (CWD 96GG)]¶	§

*CWD, chronic wasting disease; WTD, white-tailed deer. †Previously reported in Barria et al. (*15*). ‡Case 12 previously reported in Mitchell et al. (*19*). §Reportedly identical to the case of CWD in a wild reindeer in Norway (Benestad et al. [*2*]). ¶Case 47 previously reported in Mitchell et al. (*19*).

### Protein Misfolding Cyclic Amplification

#### Substrate and Seed Preparation

We homogenized transgenic mouse brains in conversion buffer by using a glass on glass manual grinder and a conversion buffer made of 1× phosphate-buffered saline (PBS), 150 mmol/L of NaCl, 1% Triton X-100, and a complete protease inhibitor cocktail (cOmplete; Roche, Mannheim, Germany) to obtain a final 10% weight-to-volume solution. We cleared the homogenized tissue by using centrifugation as described previously (*15*), aliquoted the supernatant (i.e., PMCA substrate) into 1.5 mL tubes, and stored at −80°C until used. We homogenized the CWD brain material (i.e., PMCA seed) by using 1.5-mL Eppendorf tubes and disposable polypropylene pestles that used the same buffer.

#### PMCA Procedure

We performed amplification in a programmable Q-700 sonicator attached to a microplate aluminum horn (*15*). We mixed brain homogenate CWD PMCA seeds with aliquots of PMCA substrate in a final volume of 120 µL in PCR tubes at a 1:3 ratio. We included low molecular weight heparin at 100 µg/mL (*21*) in all PMCA reactions and added EDTA to a final concentration of 6 mmol/L. To perform a comparison between samples before and after the amplification procedure, we took 19 µL of each reaction mixture before the serial cycles of sonication and incubation. Each cycle consisted of 20 s sonication (at an amplitude of 38, wattage 278–300) followed by 29 min and 40 s incubation; we repeated this procedure 96 times (48 h).

#### Proteolytic Treatment and Western Blotting

We evaluated the presence of PrP^res^ by Western blot after proteinase K treatment. We incubated 19 µL of each sample with proteinase K in a final concentration of 50 µg/mL for 1 h at 37°C in a standard thermoblock. Before loading, we mixed samples with an appropriate volume of 4× NuPAGE buffer (Invitrogen, Carlsbad, CA, USA) and boiled them at 100°C for 10 min. We loaded the samples on NuPAGE Novex (Fisher Scientific; Loughborough, United Kingdom) 10% Bis-Tris gels (1.0 mm, 10 wells) and subjected them to electrophoresis at 200 volts for 55 min. We transferred proteins to a polyvinylidene difluoride membrane by using 800 mA for 60 min (*15*) and blocked membranes with 2% milk for 1 h. We determined accumulated human PrP^res^ on the basis of the specific immunoreactivity of the 3F4 monoclonal antibody (mAb) diluted 1/10,000 (Millipore; Watford, United Kingdom). We detected CWD PrP by stripping the Western blots (Thermo Fisher, Bleiswijk, Netherlands) and reprobing them with a 6H4 antibody diluted 1/40,000 (Prionics; Schlieren, Switzerland). We used ECL antimouse IgG, peroxide-linked species-specific F(ab′)2 fragment from sheep (GE Healthcare Life Sciences; Little Chalfont, United Kingdom) as a secondary antibody diluted 1/25,000. We developed membranes by chemiluminescent detection using ECL Prime (GE Healthcare Life Sciences) and acquired digital images by using an XRS Bio-Rad system (Bio-Rad Laboratories, Hercules, CA, USA) with a CCD camera.

### Criteria for Positivity

The criterion for conversion was 3F4 antibody detection of a pattern of 3 clear protease-resistant bands of the expected electrophoretic mobility and ratio for PrP^res^ in the amplified sample after a defined period of Western blot image capture. The triplet pattern had to be absent from the unamplified sample tested under the same conditions analyzed in parallel.

### Precipitation of Insoluble PrP

We incubated brain homogenate from white-tailed deer and reindeer CWD specimens by using 20% sarkosyl (diluted in PBS) for 10 min at room temperature. We subjected samples to centrifugation for 1 h at 100,000 × *g* at 4°C as described previously (*21*). After centrifugation, we discarded the supernatant and washed the pellet with PBS followed by a second centrifugation (100,000 × *g* for 1 h at 4°C). We resuspended the washed pellets directly in PMCA substrate before using them in a single round of amplification.

## Results

### Determination of Total PrP in Cervid CWD Specimens

**Figure 1:**
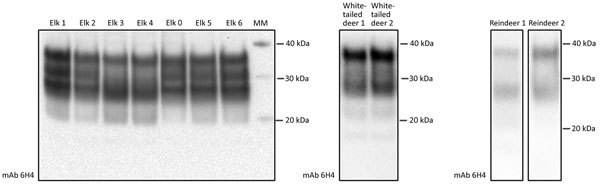
Western blot analysis showing detectable levels of prion protein in the chronic wasting disease–affected cervid brain specimens used to evaluate the susceptibility of the human prion protein (PrP) to conversion by chronic wasting disease prions. We analyzed brain homogenate derived from elk, white-tailed deer, and reindeer specimens by using Western blot to evaluate levels of total PrP. We subjected 2 μL of each 10% brain homogenate sample to Western blot and assessed detection of total PrP by mAb 6H4. We performed 3 technical repeats with similar results; a representative Western blot is shown. Reference molecular markers have been included. mAb, monoclonal antibody.

**Figure 2:**
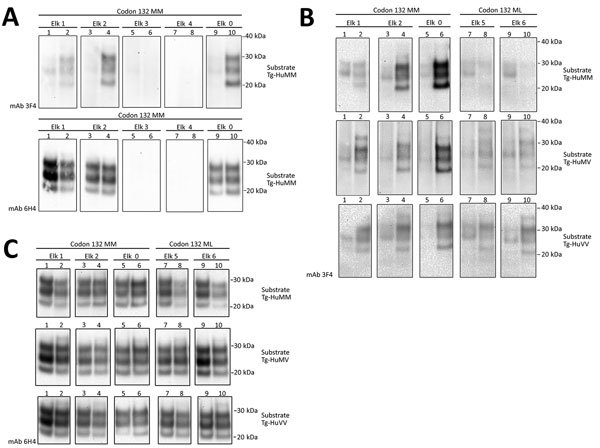
Evaluation of the in vitro conversion of human prion protein (PrP) seeded with the misfolded, disease-associated prion protein form present in chronic wasting disease (CWD)–affected elk brain samples. Western blot analysis for PrP with odd and even number lanes showing reaction mixtures before and after protein misfolding cyclic amplification. A) We incubated 5 elk CWD specimens (elk 0–4) homozygous for *Prnp* codon 132 methionine (MM) in Tg-HuMM brain substrate (diluted 1:3) and subjected them to a single round of protein misfolding cyclic amplification followed by proteinase K digestion. We performed Western blot analysis by using the mAb 3F4 (for the detection of human protease-resistant prion protein [PrP^res^]) and 6H4 (detection of CWD PrP^res^ and human PrP^res^). B) We used a panel of 3 humanized transgenic substrates (Tg-HuMM, Tg-Hu-MV, and Tg-HuVV) to evaluate the susceptibility of the human PrP to conversion. We assessed 3 CWD elk seeds of the132 MM genotype and 2 of the 132 methionine–leucine (ML) genotype. We detected conversion of the human PrP by CWD prions by using the mAb 3F4 after proteinase K digestion. C) We detected total PrP^res^ by using Western blot with mAb 6H4. The elk specimen previously reported (*15*) is designated elk 0. We performed >5 repeats for the amplification of elk CWD 132 MM seeds and >3 for the 132 ML specimens with similar results. Reference molecular markers have been included. Molecular mass of electrophoretic markers is given. mAb, monoclonal antibody; Tg-HuMM, humanized transgenic *PRNP* codon 129 homozygous methionine; Tg-HuMV, humanized transgenic methionine/valine; Tg-HuVV, humanized transgenic valine/valine.

**Figure 3:**
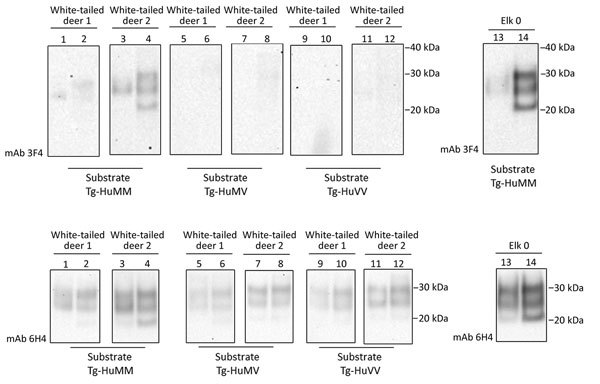
Evaluation of the in vitro conversion of human prion protein (PrP) seeded with the misfolded, disease-associated prion protein form present in chronic wasting disease (CWD)–affected white-tailed deer brain samples. We incubated 2 white-tailed deer CWD brain homogenates, derived from 2 affected animals (white-tailed deer 1 and 2), in a panel of 3 humanized transgenic substrates (Tg-HuMM, Tg-HuMV, and Tg-HuVV) and subjected them to a single round of protein misfolding cyclic amplification (PMCA) followed by proteinase K digestion. We diluted CWD brain homogenate 1:3 in PMCA substrate and performed Western blot analysis by using the mAb 3F4 (for the detection of human protease-resistant prion protein [PrP^res^]) and mAb 6H4 (for detection of CWD PrP^res^ and human PrP^res^). We incorporated the elk specimen designated elk 0 as a control. We performed >3 repeats for the amplification white-tailed deer CWD 1 and 2 specimens with similar results. Reference molecular markers have been included. Molecular mass of electrophoretic markers is given. Odd and even number lanes show reaction mixtures before and after PMCA. mAb, monoclonal antibody; Tg-HuMM, humanized transgenic *PRNP* codon 129 homozygous methionine; Tg-Hu-MV, humanized transgenic methionine/valine; Tg-HuVV, humanized transgenic valine/valine.

**Figure 4:**
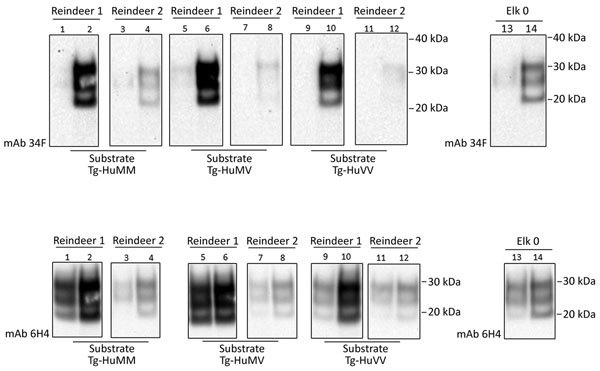
Evaluation of the in vitro conversion of human prion protein (PrP) seeded with the misfolded, disease-associated prion protein form present in chronic wasting disease (CWD)–affected reindeer brain samples. We incubated 2 reindeer CWD specimens (reindeer 1 and 2) in a panel of 3 humanized transgenic substrates (Tg-HuMM, Tg-HuMV, and Tg-HuVV) and subjected them to a single round of protein misfolding cyclic amplification (PMCA). We diluted PMCA seeds 3 times in fresh PMCA substrate (dilution factor 1:3) and evaluated PMCA reactions for the presence of protease-resistant prion protein (PrP^res^) by proteinase K digestion. We performed Western blot analysis by using mAb 3F4 (for the detection of human PrP^res^) and mAb 6H4 (for detection of CWD PrP^res^ and human PrP^res^). We incorporated the elk specimen designated elk 0 as a control. We performed >5 repeats for the amplification of reindeer 1 and 2 specimens. Reference molecular markers have been included. Molecular mass of electrophoretic markers is given. Odd and even number lanes show reaction mixtures before and after PMCA. mAb, monoclonal antibody; Tg-HuMM, humanized transgenic *PRNP* codon 129 homozygous methionine; Tg-Hu-MV, humanized transgenic methionine/valine; Tg-HuVV, humanized transgenic valine/valine.

We first characterized the CWD brain tissues for the presence of total PrP and PrP^res^ by using mAb 6H4. We detected similar levels of total PrP by using Western blotting among the elk specimens analyzed (Figure 1). However, we did not find readily detectable levels of PrP^res^ in all the samples; we detected PrP^res^ in 3 of the 5 elk specimens of the 132 MM genotype and both of the 132 ML samples (Figure 2, panels A and C). White-tailed deer samples showed similar expression levels of total PrP in the 2 specimens analyzed, but PrP^res^ levels were low (Figures 1, 3). We also confirmed total PrP in the reindeer specimens, with a robust detection of PrP^res^ in 1 of the available samples (reindeer 1) but low levels in the other specimen (reindeer 2) (Figures 1, 4).

### In vitro Conversion of Human PrP by Cervid Prions

#### Elk (*C. Canadensis*)

We then performed a single round of PMCA, incubating the *PRNP* codon 132 MM elk CWD seeds in humanized transgenic 129 MM mouse brain substrate. Only those elk CWD samples that had readily detectable PrP^res^ (as detected by 6H4 mAb) were able to produce human PrP^res^ (detectable by the 3F4 mAb) after Western blot and proteinase K treatment, consistent with CWD PrP^res^ playing a direct role in the misfolding process (Figure 2, panel A).

We then addressed the role of the human *PRNP* codon 129 and the cervid *PRNP* codon 132 polymorphisms in the conversion of human PrP^C^. To seed PMCA reactions, we used CWD brain homogenates from 132 MM and 132 ML elk with comparable quantities of CWD PrP^res^ (Figure 2, panel C) and then used humanized transgenic mouse substrates of the 3 possible *PRNP* codon 129 genotypes (129 MM, 129 MV, and 129VV). Each of the CWD methionine homozygous (132 MM) samples resulted in human PrP^res^ formation when used to seed the matched humanized substrate (129 MM). The heterozygous elk seeds did not result in any detectable conversion of the humanized 129 MM PrP substrate. When we incubated these same CWD brain homogenates with the heterozygous (129 MV) and homozygous (129VV) humanized substrates, we observed low levels of human PrP^res^ formation but no obvious difference in efficiency between the CWD 132MM and CWD 132ML samples in the 129VV substrate (Figure 2, panel B).

#### White-tailed Deer (*O. virginianus*)

We performed similar analyses to determine the competence of white-tailed deer CWD to convert the human PrP (Figure 3). To maintain consistency, we homogenized the 2 available CWD white-tailed deer specimens at 10% (weight/volume) and then normalized by volume (seed/substrate) to seed the PMCA reactions. Detection of CWD PrP^res^ by 6H4 antibody revealed that the levels of PrP^res^ in the unamplified samples were not equivalent to the elk CWD specimens used for PMCA (Figure 2, panel C; Figure 3). However, 1 of the analyzed specimens showed some conversion of the humanized 129 MM PrP substrate, although PrP^res^ formation was undetectable in the heterozygous and valine homozygous substrate with the 3F4 antibody (Figure 3). These results suggest a higher degree of molecular compatibility of the *PRNP* codon 129 MM human genotype and CWD PrP^Sc^, consistent with what we observed in most of the elk CWD 132 MM specimens.

#### Reindeer (*R. tarandus*)

**Figure 5:**
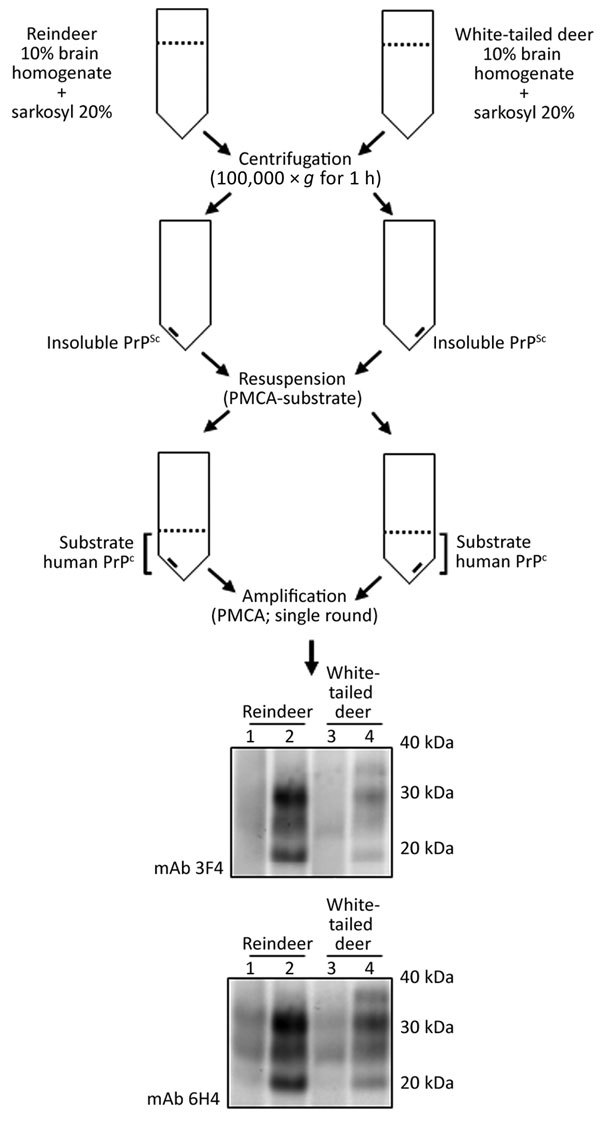
Schematic representation of the partial purification of misfolded, disease-associated prion protein from chronic wasting disease (CWD)–affected deer brain specimens and its continued ability to seed the conversion of human prion protein (PrP) during protein misfolding cyclic amplification (PMCA) reactions. We normalized PrP, partially purified by detergent insolubility from reindeer and white-tailed deer CWD specimens, by using protease-resistant prion protein (PrP^res^) and subjected PrP to a single round of PMCA in humanized transgenic *PRNP* codon 129 homozygous methionine. We performed Western blot analysis by using mAb 3F4 (for detection of human PrP^res^) and mAb 6H4 (for detection of CWD PrP^res^ and human PrP^res^). Molecular mass of electrophoretic markers is given. Odd and even number lanes show reaction mixtures before and after PMCA. mAb, monoclonal antibody; PMCA, protein misfolding cyclic amplification; PrP^c^, normal isoform of the prion protein; PrP^sc^, disease-associated isoform of the prion protein.

Natural cases of CWD in reindeer have been detected in Norway, but North American reindeer were previously shown to be experimentally susceptible to white-tailed deer CWD by the oral route (*18*). We tested samples from these 2 experimentally infected reindeer (*18*) (Figure 4). Both reindeer specimens were capable of converting the humanized 129 MM PrP substrate, although different amounts of CWD PrP^res^ were detected by the 6H4 antibody in the frozen samples. Densitometry analysis suggested that 1 specimen (from reindeer 2) had roughly one tenth of the PrP^res^ of the other sample, showing that the reaction with small amounts of reindeer PrP^res^ are able to convert the humanized substrate. The *PRNP* codon 129 MV and VV genotype substrates also were readily susceptible to conversion by the reindeer seeds (Figure 4). Seeding efficiency of reindeer CWD was maintained when the seeding material was normalized by using semipurified PrP^res^ (Figure 5), arguing against the possibility that the apparent enhanced seeding potential of reindeer CWD simply reflects the increased abundance of PrP^res^ in reindeer samples or was a result of conversion of endogenous reindeer seed–associated PrP^C^.

## Discussion

Characterization of the transmission properties of CWD and evaluation of their zoonotic potential are important for public health purposes. Given that CWD affects several members of the family *Cervidae*, it seems reasonable to consider whether the zoonotic potential of CWD prions could be affected by factors such as CWD strain, cervid species, geographic location, and *Prnp*–*PRNP* polymorphic variation. We have previously used an in vitro conversion assay (PMCA) to investigate the susceptibility of the human PrP to conversion to its disease-associated form by several animal prion diseases, including CWD (*15*,*16*,*22*). The sensitivity of our molecular model for the detection of zoonotic conversion depends on the combination of 1) the action of proteinase K to degrade the abundant human PrP^C^ that constitutes the substrate while only N terminally truncating any human PrP^res^ produced and 2) the presence of the 3F4 epitope on human but not cervid PrP. In effect, this degree of sensitivity means that any human PrP^res^ formed during the PMCA reaction can be detected down to the limit of Western blot sensitivity. In contrast, if other antibodies that detect both cervid and human PrP are used, such as 6H4, then newly formed human PrP^res^ must be detected as a measurable increase in PrP^res^ over the amount remaining in the reaction product from the cervid seed. Although best known for the efficient amplification of prions in research and diagnostic contexts, the variation of the PMCA method employed in our study is optimized for the definitive detection of zoonotic reaction products of inherently inefficient conversion reactions conducted across species barriers. By using this system, we previously made and reported the novel observation that elk CWD prions could convert human PrP^C^ from human brain and could also convert recombinant human PrP^C^ expressed in transgenic mice and eukaryotic cell cultures (*15*).

A previous publication suggested that mule deer PrP^Sc^ was unable to convert humanized transgenic substrate in PMCA assays (*23*) and required a further step of in vitro conditioning in deer substrate PMCA before it was able to cross the deer–human molecular barrier (*24*). However, prions from other species, such as elk (*15*) and reindeer affected by CWD, appear to be compatible with the human protein in a single round of amplification (as shown in our study). These observations suggest that different deer species affected by CWD could present differing degrees of the olecular compatibility with the normal form of human PrP.

The contribution of the polymorphism at codon 129 of the human PrP gene has been extensively studied and is recognized as a risk factor for Creutzfeldt-Jakob disease (*4*). In cervids, the equivalent codon corresponds to the position 132 encoding methionine or leucine. This polymorphism in the elk gene has been shown to play an important role in CWD susceptibility (*25*,*26*). We have investigated the effect of this cervid *Prnp* polymorphism on the conversion of the humanized transgenic substrate according to the variation in the equivalent *PRNP* codon 129 polymorphism. Interestingly, only the homologs methionine homozygous seed–substrate reactions could readily convert the human PrP, whereas the heterozygous elk PrP^Sc^ was unable to do so, even though comparable amounts of PrP^res^ were used to seed the reaction. In addition, we observed only low levels of human PrP^res^ formation in the reactions seeded with the homozygous methionine (132 MM) and the heterozygous (132 ML) seeds incubated with the other 2 human polymorphic substrates (129 MV and 129 VV). The presence of the amino acid leucine at position 132 of the elk *Prnp* gene has been attributed to a lower degree of prion conversion compared with methionine on the basis of experiments in mice made transgenic for these polymorphic variants (*26*). Considering the differences observed for the amplification of the homozygous human methionine substrate by the 2 polymorphic elk seeds (MM and ML), reappraisal of the susceptibility of human PrP^C^ by the full range of cervid polymorphic variants affected by CWD would be warranted.

In light of the recent identification of the first cases of CWD in Europe in a free-ranging reindeer (*R. tarandus*) in Norway (*2*), we also decided to evaluate the in vitro conversion potential of CWD in 2 experimentally infected reindeer (*18*). Formation of human PrP^res^ was readily detectable after a single round of PMCA, and in all 3 humanized polymorphic substrates (MM, MV, and VV). This finding suggests that CWD prions from reindeer could be more compatible with human PrP^C^ generally and might therefore present a greater risk for zoonosis than, for example, CWD prions from white-tailed deer. A more comprehensive comparison of CWD in the affected species, coupled with the polymorphic variations in the human and deer *PRNP–Prnp* genes, in vivo and in vitro, will be required before firm conclusions can be drawn. Analysis of the *Prnp* sequence of the CWD reindeer in Norway was reported to be identical to the specimens used in our study (*2*). This finding raises the possibility of a direct comparison of zoonotic potential between CWD acquired in the wild and that produced in a controlled laboratory setting. (Table).

The prion hypothesis proposes that direct molecular interaction between PrP^Sc^ and PrP^C^ is necessary for conversion and prion replication. Accordingly, polymorphic variants of the PrP of host and agent might play a role in determining compatibility and potential zoonotic risk. In this study, we have examined the capacity of the human PrP^C^ to support in vitro conversion by elk, white-tailed deer, and reindeer CWD PrP^Sc^. Our data confirm that elk CWD prions can convert the human PrP^C^, at least in vitro, and show that the homologous *PRNP* polymorphisms at codon 129 and 132 in humans and cervids affect conversion efficiency. Other species affected by CWD, particularly caribou or reindeer, also seem able to convert the human PrP. It will be important to determine whether other polymorphic variants found in other CWD-affected *Cervidae* or perhaps other factors (*17*) exert similar effects on the ability to convert human PrP and thus affect their zoonotic potential.
